# Exploring the Characteristics of an Aroma-Blending Mixture by Investigating the Network of Shared Odors and the Molecular Features of Their Related Odorants

**DOI:** 10.3390/molecules25133032

**Published:** 2020-07-02

**Authors:** Anne Tromelin, Florian Koensgen, Karine Audouze, Elisabeth Guichard, Thierry Thomas-Danguin

**Affiliations:** 1 Centre des Sciences du Goût et de l’Alimentation, AgroSup Dijon, CNRS, INRAE, Université Bourgogne Franche-Comté, F-21000 Dijon, France; koensgen.florian@gmail.com (F.K.); elisabeth.guichard@inrae.fr (E.G.); thierry.thomas-danguin@inrae.fr (T.T.-D.); 2 Université de Paris, INSERM UMR-S1124, F-75006 Paris, France; karine.audouze@u-paris.fr

**Keywords:** odorants, odor notes, configural mixture, network, statistical analysis, pharmacophore

## Abstract

The perception of aroma mixtures is based on interactions beginning at the peripheral olfactory system, but the process remains poorly understood. The perception of a mixture of ethyl isobutyrate (Et-iB, strawberry-like odor) and ethyl maltol (Et-M, caramel-like odor) was investigated previously in both human and animal studies. In those studies, the binary mixture of Et-iB and Et-M was found to be configurally processed. In humans, the mixture was judged as more typical of a pineapple odor, similar to allyl hexanoate (Al-H, pineapple-like odor), than the odors of the individual components. To explore the key features of this aroma blend, we developed an in silico approach based on molecules having at least one of the odors—strawberry, caramel or pineapple. A dataset of 293 molecules and their related odors was built. We applied the notion of a “social network” to describe the network of the odors. Additionally, we explored the structural properties of the molecules in this dataset. The network of the odors revealed peculiar links between odors, while the structural study emphasized key characteristics of the molecules. The association between “strawberry” and “caramel” notes, as well as the structural diversity of the “strawberry” molecules, were notable. Such elements would be key to identifying potential odors/odorants to form aroma blends.

## 1. Introduction

The first step of odor detection is the interaction between the odorants and the olfactory receptors (ORs) in the nose [1]. The perception of an odor’s quality is a result of combinatorial coding [2], whereby an odorant can interact with several ORs, while ORs can be activated by several structurally diverse odorants. Despite advances in the understanding of olfactory perception, olfactory coding remains poorly understood [3,4], especially in the case of a mixture of odorants. Still, odors perceived in our environment are mainly the result of mixtures of odorants [5].

It has been theorized and experimentally confirmed that the olfactory processing of a mixture of odorants can produce two types of percepts: (i) heterogeneous percepts in which the specific odor qualities of several individual odorants can be identified within the mixture; or (ii) homogeneous percepts in which a single odor is perceived from the mixture [5,6].

A homogeneous percept can result from a configural processing of the mixture or from complete overshadowing (or masking) [5,7]. Odor blending occurs if a mixture of molecules A and B carrying different odors is configurally processed and thus perceived to have a specific new odor, distinct from the odors of each component of the AB mixture [8]. In summary, a blending mixture percept can be represented as AB ≠ A + B.

It is now accepted that the odor perception results from interactions occurring between the peripheral olfactory system and the brain [3,9]. Nevertheless, the precise pathway(s) involved in the homogeneous perception of odor mixtures remains poorly understood [6,10]. To date, they are mainly target approaches, which concern the interactions of odorants at the OR and olfactory sensory neuron levels [11,12,13,14,15,16,17,18,19,20,21,22], evoking odorants that are involved as agonists/antagonists of their biological targets.By contrast, we focused on a ligand approach, which is complementary to the target approach. In the context of aroma blending, our approach consisted of considering a set of odorants, whose selection was based on aroma blending and whose perceptual and configural characteristics have been previously carefully investigated in studies performed with animals [23,24] and humans [25,26,27]. These studies repeatedly showed that the perception of a mixture of ethyl isobutyrate (Et-iB), which has a strawberry-like odor (STR), and ethyl maltol (Et-M), which has a caramel-like odor (CAR), is processed by the olfactory system in a configural way. In humans, the mixture (Et-iB + Et-M) was investigated in comparison with a reference, namely, allyl hexanoate (Al-H), which has a pineapple-like odor (PNA), and it was demonstrated that the mixture has an odor close to this reference. In addition, the binary mixture was judged as having an odor more typical of pineapple than of the individual components [25].

The aim of the present work was to identify the characteristics of odorants carrying the same odor notes as those of the mixture “Et-iB + Et-M ≡ Al-H”, i.e., in terms of the odor notes STR + CAR ≡ PNA, with the objective of helping to understand aroma blending perception. To explore the key features of these odorants, we extracted from a large database of odorants (3508 odorants, 251 odor notes [28]) all of the molecules having at least one of the odors—STR, CAR or PNA—in their odorant description (henceforth called STR/CAR/PNA odorants). The obtained dataset, called “StCaPi-set”, included 293 molecules and 112 odors.

We adopted a systematic, detailed approach without a priori hypotheses to explore the odorous and molecular properties of the StCaPi-set odorants along two axes. The first axis is in line with studies that highlight the significance of the biological function of odorants, that is, their odor, to understand odorant discrimination [29,30]. From this perspective, on the basis on our recent work on the analysis of a large odorant database [28], we applied the notion of a “social network” to all the odor notes shared by the STR/CAR/PNA odorants in the StCaPi-set. In social sciences, such a network is used to study relationships among individuals. We followed a similar approach to describe the network of odor notes linked to STR, CAR and/or PNA.

The second axis concerns the properties of the molecules and the spatial distribution of the molecular features in the STR/CAR/PNA odorants in the StCaPi-set. Assuming that combinations of activated ORs encode odor qualities and that molecules sharing the same odorant quality possess common structural molecular properties [31,32], our assumption was that molecules having strawberry, caramel or pineapple odors should have structural features specific to each odor. Moreover, the odor blending “STR + CAR ≡ PNA” suggests that STR/CAR/PNA odorants could also share common structural features. We developed a statistical analysis method based on several molecular descriptors; additionally, we applied a pharmacophore approach to explore the structural similarities between the STR/CAR/PNA molecules.

Thus, the purpose of the present paper was to improve our understanding of the complex issue of aroma blending mixture perception by looking for key characteristics of the aroma blend STR + CAR ≡ PNA. The results highlight several key characteristics of the odorants, revealing peculiar links between the odors STR, CAR and PNA, as well as specific chemical properties associated with each subset of odorants, who additionally have common spatial distributions for several chemical features.

## 2. Results

### 2.1. Odorants, Odor Descriptions Involved in the Mixture and Data Organization

We selected molecules from a large database, previously used to identify links between odor notes and odorants by multivariate statistical analysis [28]. This database, called FB-3508, includes 3508 odorants and 251 odor notes as a binary matrix and was built from the 9th version of the commercially available Flavor-Base [33]. The selection of the molecules was based on the occurrence of the simple odor notes “strawberry” (STR), “caramellic” (CAR) and “pineapple” (PNA) in their odor description. “Simple odor” notes are distinct from complex odors based on “strawberry” and “pineapple”. For example, molecules described as “cooked/jammy strawberry/pineapple” possess specific aromas that differ from those of fresh fruits. Thus, we have chosen not to consider such complex odor notes as STR or PNA.

Additionally, we included two molecules that do not strictly meet the selection criteria:

- Et-iB was included, since this molecule is one component of the target blending mixture. It is not described by “strawberry” in Flavor-Base (“*sweet, ethereal, fruity rum like odor and taste; apple notes*”), but is in other databases (e.g., FlavorDB “*rubber, alcoholic, ethereal, strawberry, sweet, fusel, fruity, rummy*” [34]);
- Strawberry furanone, described as “*fruity, caramelized pineapple-strawberry odor & taste; roasted*” was included because this molecule is a key contributor to the aroma of strawberry [35].

Hence, the obtained, referred to as StCaPi-set, encompasses 293 odorant molecules and 112 odors as a binary matrix (Table S1). This matrix has been used for the study of odor notes and links between odor notes; it includes 23, 153, and 129 STR, CAR and PNA molecules, respectively.

Moreover, five of the odorants are described as mixtures of isomers, and each isomer should be considered a specific molecular structure for the generation of pharmacophores:

- Amyl keto dioxane (CAR molecule), 2 isomers: 5-(or 6-)pentyl-1,4-dioxan-2-one);
- Butylketodioxane (CAR molecule), 2 isomers: 5-(or 6-)butyl-1,4-dioxan-2-one);
- Tetramethylethylcyclohexenone (CAR molecule), 2 isomers: 5-ethyl-2,3,4,5 (and 3,4,5,6)-tetramethyl-2-cyclohexen-1-one;
- Isobutyl 4-decenoate (PNA molecule), 2 isomers: cis- and trans-isobutyl 4-decenoate;
- 8-ocimenyl acetate (PNA molecule), 4 isomers due to 2 double bonds, of which only 2 are described with a “pineapple” note in Flavor-Base: (Z2,E5)-2,6-dimethylocta-2,5,7-trien-1-yl acetate, (E2,Z5)-2,6-dimethylocta-2,5,7-trien-1-yl acetate.

Consequently, 298 structures were considered in this study. However, the StCaPi-set should not be taken as a whole for structural analysis, and it was divided into subsets according to the odor associations.

These subsets are the following (Table S1):

- Three “simple odor” subsets: s-STR (10 molecules), s-CAR (146 molecules) and s-PNA (126 molecules). The molecules of this subset carry one of the three odors of the blend. Molecules described with several notes -STR, CAR or PNA- do not belong in these subsets;
- Three “true odor” subsets: t-STR, t-CAR and t-PNA. The “true odor” subsets are included in the s-STR, s-CAR and s-PNA subsets, respectively, and each of them contain seven molecules. All compounds that were additionally described by any other note (except “fruity”) were excluded; nevertheless, this condition was difficult to obtain for s-STR molecules. The list and the odor description of the molecules in the “true odor” subsets are reported in Table 1;
- Two subsets of “mixed odors” encompass molecules with two reference odor notes: STR-CAR (nine molecules) and STR-PNA (four molecules). There is no CAR-PNA subset because only one molecule, alpha-furfuryl pentanoate, has these two odors (“*fruity-pineapple-apple, caramellic odor; ripe pineapple-apple fruity taste*”);
- One subset “EXP” encompasses the three molecules involved in the experimental blending mixture [36]: Et-iB (ethyl isobutyrate) and Et-M (ethyl maltol), which belong to the subsets t-CAR, and Al-H (allyl hexanoate, “*fatty, fruity, winey-pineapple like odor”*).

### 2.2. Network of Odors Shared by the Aroma-Blending Mixture

To study the associations between odor notes, we calculated the symmetric square of the two-way cross-tabulations (cooccurrence matrix) obtained from the transposed binary matrix of StCaPi-set, in which the odor notes are the observations (rows) and the odorants are the variables (columns). The achieved symmetric square matrix provides the number of odorants in which the two odor notes appear together in the odorant description for each possible pair of odor notes. This number of cooccurrences is called the “frequency of association” between the two odor notes.

**Figure 1:**
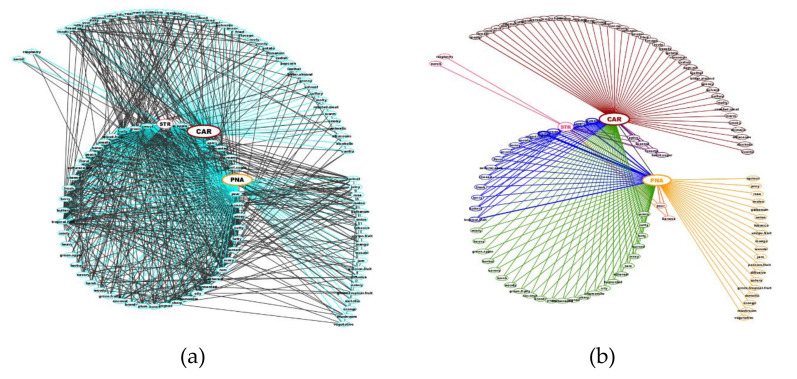
Network of odor notes in the StCaPi-set: (**a**) the whole network of odors (750 pairs): in blue, 174 links linked to STR, CAR or PNA, and in gray, 576 links between the other odor notes. (**b**) The network of pairs involving STR, CAR or PNA (172 pairs); Level L1 links to STR (2 odors) are shown in pink, L1 links to CAR (43 odors) are shown in brown, and L1 links to PNA (19) are shown in pale orange. Level L2 links: 15 odorants link STR-CAR-PNA (in blue), 4 odorants link STR-CAR (in purple), 2 odorants link STR-PNA (in orange-pink), and 24 odorants link CAR-PNA (in green).

By stacking the cooccurrence matrix and removing the diagonal elements, we obtained 12,432 pairs of odor notes. Excluding the duplicate pairs and the 10,932 pairs with cooccurrences of zero resulted in a list of 750 pairs (Figure 1).

To describe the network of odors linked to STR, CAR and/or PNA, we applied the notion of a “social network”, which is used in social sciences to study the relationships among individuals.

Therefore, if two odor notes co-exist in the description of one or several odorants, the odor notes are “linked at level L1”. The number of links between two odor notes is the number of molecules described with those two odor notes. If no Level L1 link exists, but the two odor notes are both associated with the same third odor note, they are linked through a “bridge” (one “intermediate note”, or two ties); in other words, they are “linked at Level L2”. In the StCaPi-set, the STR, CAR and PNA odors occur 23, 153 and 129 times, respectively, representing 0.7%, 4.4% and 3.7% of the 3508 odorants in the previously analyzed database.

The STR, CAR and PNA odors are linked to 25, 87 and 62 other notes, respectively, while there are 578 links between all other odors (Figure 1a).

The examination of the links between STR, CAR and PNA at Level L1 revealed nine STR-CAR cooccurrences, four STR-PNA cooccurrences, and only one CAR-PNA cooccurrence.

CAR and PNA are specifically linked to 43 and 19 odor notes, respectively. Conversely, only two notes (neroli and raspberry) are connected only to STR. All other odors linked to STR are also linked to CAR and PNA (15 L2 links), to CAR (four L2 links) and to PNA (two L2 links, pear and banana). Finally, CAR and PNA are linked at Level L2 by 24 odor notes (Figure 1b).

L1 and L2 links could be important elements in the formation of odor blending, and several observations were made based on examining the number of links between odor notes:

- STR is quite infrequent (STR molecules represent less than 1% of the whole FB-3508 database), and STR is associated with 25 other odors. In fact, STR is never the sole descriptor. Approximately 40% of the occurrences of STR show cooccurrence with CAR, which is the most frequent association, except for the general notes fruity (16 cooccurrences) and sweet (11 cooccurrences). In addition, despite their common fruity odor, STR cooccurs only four times with pineapple;
- CAR and PNA cooccur in just one molecule described in the Flavor-Base 9th Ed., alpha-furfuryl pentanoate, which is described as “*fruity-pineapple-apple, caramellic odor; ripe pineapple-apple fruity taste*”.

When examining the involved odor notes and the links between them, several observations can be made. The most frequent associations with “strawberry” are the odor notes “fruity” and “sweet” (which cooccur with 73.91% and 52.17% of “strawberry” occurrences, respectively), followed by “caramellic (39.13%), and then “pineapple” and “apple” (both cooccur 17.4% of “strawberry” occurrences). Conversely, neither “ethereal” nor “rum” are linked with “strawberry” in the StCaPi-set, but interestingly, “ethereal” and “rum” are bridges between “caramellic” and “pineapple”. Therefore, although the “strawberry” odor note of Et-iB is difficult to unequivocally consider, this odorant clearly belongs to the part of the network occupied by the odor notes linked to both “caramellic” and “pineapple”.

Another important consideration concerning the STR note is that at least one other odor note is present in the odor description of each molecule with the STR note. The sole exception in the StCaPi-set is phenylpropyl isovalerate, for which only the “fruity” note was considered. However, the entire description of this molecule is “*fruity (strawberry-prune) odor; sweet “preserve” like taste*”, which means “strawberry” is associated with “prune”. However, “prune” occurs less than five times in the Flavor-Base, which was the minimum frequency threshold selected, and “prune” was not considered in the analysis.

The nature of the associations between the CAR and PNA notes differs from those of STR. Indeed, both “caramellic” and “pineapple” are used alone or in association with “fruity” and/or “sweet” (Table S1).

### 2.3. Molecular Structure Exploration

We considered the chemical structures of the 293 odorants. Five odorants are mixtures of isomers, resulting in a total of 298 molecular structures (cf Materials and Methods). We explored the structures of the odorants distributed into the following subsets (Table S1):

- Three subsets “simple odor”: s-STR (n = 10), s-CAR (n = 146), and s-PNA (n = 126);
- Three subsets “true odor”: t-STR, t-CAR, and t-PNA (n = 7 for each subset; Table 1);
- Two subsets “mixed odors”: STR-CAR (n = 9) and STR-PNA (n = 4).

The structural study consists of two parts. The first part addresses the issue of basic molecular properties, while the second part concerns the characterization of the 3D spatial distribution of the molecular features by the pharmacophore approach.

#### 2.3.1. Statistical Analysis of the Molecular Descriptor Values

We examined some properties of the StCaPi-set odorants using molecular descriptors to assess their overall structural characteristics [37]. We focused on five basic properties commonly involved in the biological activity of organic molecules: molecular weight (Molecular_Weight, noted MW), hydrophobicity (ALogP98), polarizability (Apol), flexibility (PHI) and polar solvent accessible surface area for each molecule using a 3D method (Molecular_3D_PolarSASA, noted “3D_PolarSASA” for simplicity). All the values are reported in Table S2. Our leading idea was that differences in the distribution of these property values could indicate various targeted modes of action of the StCaPi odorants.

The odorants alpha-furfuryl pentanoate (the only CAR-PNA molecule), Et-iB (“strawberry” component of the target blending mixture) and strawberry furanone (“*caramelized pineapple-strawberry odor*”) were not included in the statistical analysis. Nevertheless, we compared their descriptor values to those provided by the descriptive analysis. We also focused on Et-M and Al-H, which represented typical “caramellic” and “pineapple” compounds, respectively, in the target blending mixture. The descriptor values of these six molecules are reported in Table 2.

##### Descriptive Statistics

We aimed to assess the distribution of the molecular properties of StCaPi odorants according their various odor notes. For that purpose, we performed a descriptive statistical analysis for the molecular descriptor values according to the subsets “simple odors”, “true odors”, and “mixed odors”.

The statistical parameter values are reported in Table S3. The histograms of the distributions of the molecular descriptor values of the subsets are displayed in Figure S1.

The molecular properties values vary from:

- 74.079 to 256.424 for MW;
−0.776 to 6.122 for ALogP98;
- 2,479.480 to 12,302.700 for Apol;
- 0.881 to 14.388 for PHI;
- 10.286 to 187.129 for 3D_PolarSASA.

**Figure 2:**
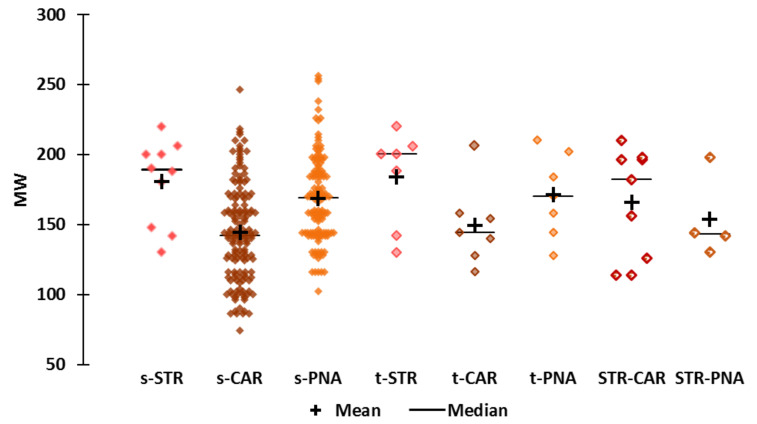
Scattergrams of the MW values by odor subset.

**Molecular Weight (Figure 2)**

The smallest molecules (MW < 110) are in the s-CAR subset (acetol, “*slightly green; weak sweet somewhat caramellic-winey note*”, MW = 74, has the smallest MW value). The largest molecules are in the s-PNA subset (decyl hexanoate, “*oily-fruity, fatty with some pineapple notes*”, MW = 256, has the largest MW value). Nevertheless, several s-CAR molecules have molecular masses higher than 200, such as benzyl disulfide (MW = 246, “*harsh, burnt-caramellic, earthy, green sulfurous odor*”) and ethyl acetylcinnamate (MW = 218, “*spicy, with caramellic fruity notes*”). The compounds in the t-CAR and t-PNA have wide ranges of MW values (116 to 206 and 128 to 210, respectively).

The molecular weights of Et-M (CAR, MW = 140) and Al-H (PNA, MW = 156) are lower than the median and mean values of the corresponding CAR and PNA subsets. The molecular weight of Et-iB (MW = 116.2) is lower than those of all the other s-STR molecules but close to that of acetonyl acetate (s-CAR, MW = 116.1; “*fermented, sour fruity-buttery, caramellic notes*”). The MW values of the nine STR-CAR molecules range from 114 to 210. Eight are cyclic molecules (five maltol derivatives and three furan derivatives) and one is a branched unsaturated acid (2-methyl-2-pentenoic acid, MW = 114, “*sweet, green, caramellic, characteristic strawberry*”).

The MW values of s-STR molecules ranged from 130 to 220; the median and mean MW values of the molecules of this subset are 189 and 180, respectively, and these are the highest median and mean MW values of the “simple odors” subsets. The smallest STR molecule, ethyl 2-methyl butyrate (“*strong, green, fruity, apple odor and taste; also some strawberry notes*”, MW = 130), belongs to the t-STR subset. The “strawberry” note of this molecule seems to be faint, as in the case of Et-iB (MW = 116.2) used for the sensory experiment [36]. Interestingly, methyl 2-methylpropanoate is the smallest s-PNA molecule (MW = 102, “*fruity, apple-pineapple-apricot-rum like odor*”).

The smallest STR-PNA molecule is isopropyl butyrate (MW = 130.2, “*strong, pineapple-strawberry & buttery odor*”), and the largest is propyl cyclohexanepropionate (MW = 198, “*strong, sweet, fruity, pineapple-peach, pear notes*”).

The MW of the unique CAR-PNA molecule, alpha-furfuryl pentanoate (MW = 182.2) is in the 3rd quartile of MW values of s-PNA molecules (169 to 188) and t-PNA (170 to 193), but is larger than the mean MW of the s-PNA and t-PNA molecules (169 and 171, respectively).

Strawberry furanone (MW = 128, *“fruity, caramelized pineapple-strawberry odor & taste; roasted”*) is one of the smallest molecules in StCaPi-set, and its molecular weight is equal to those of two CAR molecules, namely, oxoethylbutanolide (“*weak, slight caramellic, maple, burnt sugar*”) and sotolon (“*powerful caramel aroma*”).

**Figure 3:**
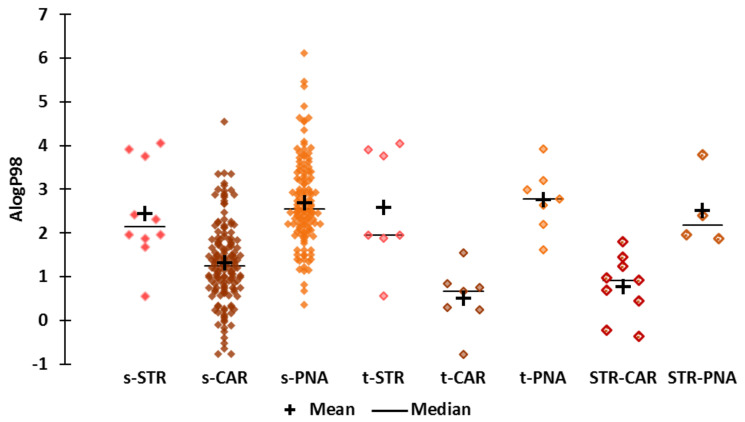
Scattergrams of the AlogP98 values by odor subset.

**Hydrophobicity (Figure 3)**

The AlogP98 values reflect the hydrophobicity of the compounds and range from −0.776 to 6.122. Not surprisingly, the less hydrophobic molecules belong to the s-CAR subset (acetol) and the more hydrophobic molecules belong to the s-PNA subset (decyl hexanoate). The t-STR molecules include mildly hydrophobic fraistone (AlogP98 = 0.558, “*fresh, sweet-fruity notes reminiscent of apple and strawberry*”) and have the same range of ALogP98 values as the s-STR molecules. The t-PNA molecules are more hydrophobic than the s-PNA subset, while the t-CAR molecules are less hydrophobic than the s-CAR subset. The least hydrophobic STR molecule is a “mixed odor” STR-CAR, hydroxymethylfuranone (ALogP98 = −0.371), which is described “*sweet, caramel, burnt sugar, roasted chicory, maltol-like*”. We classified this molecule as “STR-CAR” because of the “maltol-like” note (maltol, AlogP98 = −0.222, is described “*sweet, fruity, berry, caramellic odor; strawberry, fruity preserve-like”)*. The most hydrophobic STR molecule is naphthyl butyl ether (ALogP98 = 4.05), which is a t-STR molecule (“*sweet tenacious fruity and floral note reminiscent of raspberry and strawberry*”).

Et-M (AlogP98 = 0.301) is in the least hydrophobic quartile of the CAR molecules (−0.776 to 0.736). The ALogP98 value of Al-H (2.673) is consistent with the average for PNA molecules. Both Et-iB (AlogP98 = 1.499) and strawberry furanone (AlogP98 = 0.113) are slightly hydrophobic, with Et-iB being more hydrophobic than strawberry furanone. Their ALogP98 values are within the first quartiles of the STR and t-CAR molecules, respectively. The hydrophobicity value of alpha-furfuryl-pentanoate is within the third quartiles of STR and PNA, and is higher than the average for CAR molecules.

**Figure 4:**
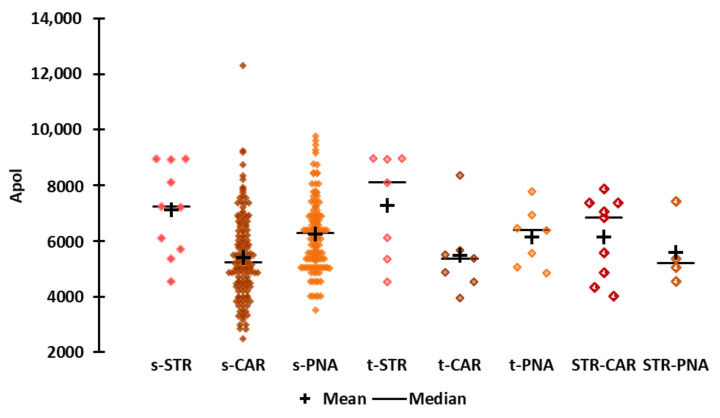
Scattergrams of the Apol values by odor subset.

**Polarizability (Figure 4)**

The STR molecules include the most polarizable molecules in the StCaPi-set, whereas the CAR molecules, and especially the t-CAR molecules, are less polarizable. The least hydrophobic compound acetol is also the least polarizable molecule of the entire StCaPi-set (Apol = 2,479). However, the most polarizable molecule is benzyl disulfide, which belongs to the s-CAR subset (Apol = 12,303, “*harsh, burnt-caramellic, earthy, green sulfurous odor*”). The least polarizable STR molecule is ethyl 2-methylbutyrate (Apol = 4,533), and the most polarizable is the t-STR molecule naphthyl butyl ether (Apol = 8963). The least and the most polarizable PNA molecules are isobutyl acetate (Apol = 4,019, “*fruity, banana-apple-pear-pineapple notes*”) and geranyl hexanoate (Apol = 9,781, “*fruity, rose-pineapple-tropical odor*”), respectively.

The polarizabilities of Et-M and Al-H are close to the medians of the CAR and PNA molecules, respectively. Et-iB and strawberry furanone have low Apol values. Et-iB is less polarizable than hydroxymethylfuranone (STR-CAR molecule, Apol = 4025, “*sweet, sugary, caramel, bread like*”), and its Apol value is within the first quartiles of the CAR and s-PNA molecules. Strawberry furanone (Apol = 4538) is among the least polarizable molecules, and its Apol value is within the first quartile of all the subsets. Conversely, alpha-furfuryl-pentanoate is among the most polarizable molecules, and its Apol value (6906) is within the third quartile of the CAR and PNA subsets.

**Figure 5:**
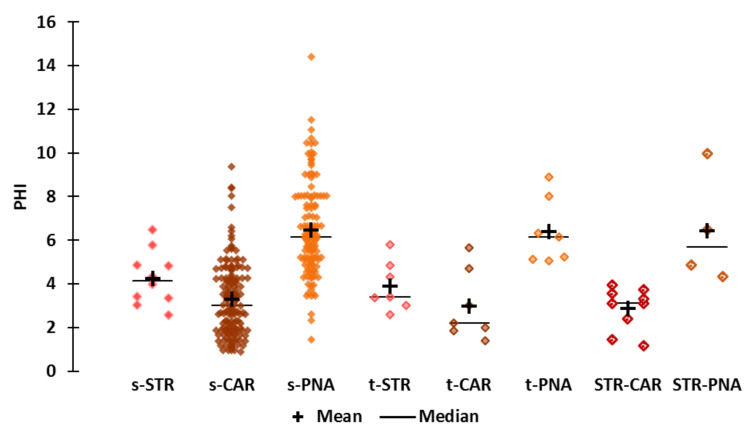
Scattergrams of the PHI values by odor subset.

**Flexibility (Figure 5)**

The flexibility of the molecule is encoded by the topological descriptor PHI (Figure 5). The PHI values range from 0.9 to 14. The CAR molecules are not very flexible, and the least flexible of which, hydroxymethylfuranone, belongs to the STR-CAR subset. The more flexible molecules belong to the PNA subset; nevertheless, piperitenone oxide has little flexibility (PHI = 1.5, “*apple, pineapple herbaceous mint odor*”). The PHI values of STR molecules are evenly distributed between the median PHI values of CAR and PNA. The least and most flexible STR molecules are ethyl methylphenylglycidate (PHI = 2.6, “*sweet, fruity-strawberry, candy-like odor*”, t-STR molecule) and isobutyl methylthiobutyrate (PHI = 6.5, “*sulfuraceous, tropical over-ripe fruity, strawberry, cream & cheese notes*”), respectively. STR-CAR compounds have very low flexibility (PHI values from 1.2 to 3.9), and STR-PNA compounds have the highest flexibility (PHI values range from 4.3 to 10); the least and most flexible molecules are isopropyl butyrate (“*strong, pineapple-strawberry & buttery odor*”), which is also the smallest STR-PNA molecule, and ethyl cis-4-decenoate (“*fruity, slight floral, pineapple-pear, peach & strawberry notes*”), respectively.

The PHI value of Et-M is within the second quartile of the CAR subset, while the PHI value of Al-H is within the third quartile of the PNA subset, confirming the low flexibility of Et-M and the good flexibility of Al-H. Et-iB is rather flexible, as reflected by its PHI value, which falls within the second quartile of the s-STR subset. The PHI value of alpha-furfuryl-pentanoate (PHI = 4.3) is within the third quartile of s-CAR but within the first quartile of s-PNA, highlighting its average flexibility. Strawberry furanone (PHI = 1.4) is the least flexible of these six molecules, and its PHI value is the same as that of sotolon (t-CAR molecule) and is within the first quartiles of the s-CAR and STR-CAR subsets.

**Figure 6:**
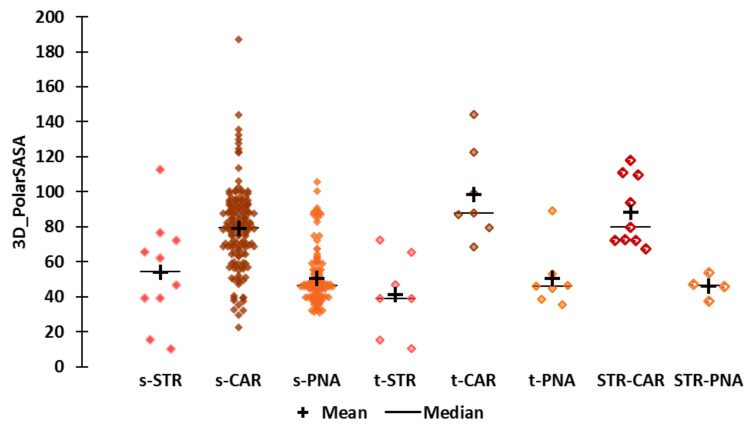
Scattergrams of the 3D_PolarSASA values by odor subset.

**Polarity (Figure 6)**

The 3D_PolarSASA values range from 10.3 to 187. The STR molecules are less polar, especially naphthyl derivatives such as naphthyl isobutyl ether (3D_PolarSASA = 10.3, “*sweet, strawberry-fruity, neroli-like*”) and naphthyl butyl ether (3D_PolarSASA = 15.4), which is the most hydrophobic STR molecule. Both of these molecules are regarded as t-STR molecules. The CAR molecules had the highest 3D_PolarSASA values, which reflect the high polarity of these compounds and is consistent with their low hydrophobicity and polarizability. However, some CAR molecules have low Polar-SASA values that are lower than or close to those of PNA molecules (22 < 3D_PolarSASA < 33). These are medium-sized (MW < 200) “fruity” molecules that are more hydrophobic, less polarizable and as flexible as the average CAR molecules (1.229 < AlogP98 < 3.606, 3000 < Apol < 8000, and 2 < PHI < 5). The 3D_PolarSASA values of the STR-CAR and STR-PNA molecules are close to those of the t-CAR and t-PNA molecules and similar to the average values of s-CAR and s-PNA, respectively, which reflects the higher polarity of the STR-CAR molecules relative to the STR-PNA molecules.

Et-M (3D_PolarSASA = 4899.3) and Al-H (3D_PolarSASA = 48) fall among the moderately polar molecules of their respective subsets. Indeed, the 3D_PolarSASA values belong to the fourth and third quartiles of the s-CAR and the s- and t-PNA subsets, respectively. The 3D_PolarSASA value of Et-iB (3D_PolarSASA = 39.4) is at the lower limit of the second quartile of s-STR and the upper limit of the third quartile of t-STR, indicating an average polarity with respect to STR molecules.

The polarity of alpha-furfuryl pentanoate is moderate compared to CAR molecules (3D_PolarSASA = 69.43 close to the upper limit of the first quartile of the CAR subsets), but high compared to PNA molecules (3D_PolarSASA in the fourth quartiles of s-PNA and t-PNA).

**Figure 7:**
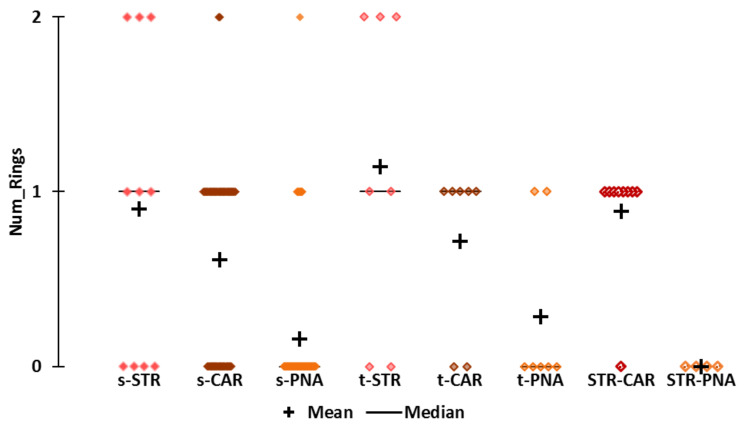
Scattergrams of the number of rings (Num_Rings) by odor subset.

**Number of rings (Figure 7)**

To better understand the structural properties that distinguish the CAR and STR-CAR subsets from other subsets, we examined the number of rings (Num Rings) in the molecular structures (Table S2). The number of rings was of particular interest because a high PHI indicates a limited degree of conformational freedom, which is commonly due to the presence of cyclic structures. We did not consider the Num_Ring descriptor for the statistical analysis (normality tests and nonparametric tests).

Not surprisingly, the s-CAR, t-CAR and STR-CAR subsets are rich in monocyclic molecules. There are five and eight monocyclic molecules in t-CAR and STR-CAR, respectively. Only one STR-CAR molecule (2-methyl-2-pentenoic acid) is acyclic, four are furans and five are maltol derivatives. Among the five cyclic CAR derivatives, there is one furan (sotolon 4,5-dimethyl-3-hydroxy-2(5H)-furanone) and one maltol (Et-M), and the three other compounds belong to different chemical families.

##### Normality Tests

We checked the normality of the descriptor value distributions for each subset using the four tests available in the XLStat package (Shapiro–Wilk, Anderson–Darling, Lilliefors and Jarque–Bera). We accepted normality when the conditions were satisfied for the four tests. According to the results of the tests, most of the descriptor values follow a normal distribution for the various subsets except (i) MW and AlogP98 for s-PNA; (ii) Apol, PHI and 3D_PolarSASA for s-PNA and s-CAR; and (iii) Polar-SASA for t-PNA. All the other variable distributions meet the tests for each subset on which they depend. However, although the normality tests did not reject the H_0_ hypothesis for the subsets that included STR molecules, this result is not significant due to the very small number of observations. Consequently, due to the disparate sizes of the subsets and because several of them do not follow a Gaussian distribution, we report here the results of a nonparametric test. Detailed results are displayed in Table S3.

##### Nonparametric Tests

We performed nonparametric Kruskal–Wallis tests for each descriptor to compare their distributions between the subsets: (i) s-STR, s-CAR and s-PNA; (ii) t-STR, t-CAR and t-PNA; (iii) s-STR subset and the two “mixed odor” subsets STR-CAR and STR-PNA.

The Kruskal–Wallis test is performed by ranking all values from the lowest to the highest regardless of the group the value is assigned to. The smallest number receives a rank of 1, while the largest number receives a rank of N, where N is the total number of values in each group. The discrepancies among the sum of the ranks are combined to create a single value named the Kruskal–Wallis statistic (K observed value). A large K (observed value) corresponds to a large discrepancy among the sum of the ranks.

As shown in Table 3, the computed p-value is lower than the significance level alpha = 0.05, leading to the rejection of the null hypothesis. Thus, there are significantly different distributions of the molecular descriptor values between some subsets.

The detailed statistical results of the pairwise comparisons using Dunn’s procedure are available in Table S4**,** and the results are summarized in Table 4.

Multiple pairwise comparisons between odor subsets using Dunn’s procedure collectively categorize STR and PNA subsets for all molecular descriptor values, except PHI values. Moreover, t-CAR and STR-CAR are in the same group considering all descriptor values except 3D_PolarSASA, for which groupings had not been performed. Nevertheless, pairwise comparisons do not reveal significant differences between t-CAR and STR-CAR subsets for Polar-SASA values.

#### 2.3.2. Pharmacophore Approach

##### Pharmacophore Generation

Odor perception is based on olfactory coding, and an odorant can activate several unknown ORs. Ligand-based pharmacophore modeling is a key computational strategy that is particularly useful when targets of active molecules are unknown. Thus, the pharmacophore approach is of great interest because it is a qualitative method that considers the intrinsic properties of the odorants [38,39]. Such an approach is well suited to the issue of odor perception at the peripheral step.

A pharmacophore is defined as a specific 3D arrangement of structural features that is common in active molecules interacting with a target receptor in a specific binding site [39]. The IUPAC definition was refined as follows [40]: “*A pharmacophore is the ensemble of steric and electronic features that is necessary to ensure the optimal supramolecular interactions with a specific biological target structure and to trigger (or to block) its biological response. A pharmacophore does not represent a real molecule or a real association of functional groups, but a purely abstract concept that accounts for the common molecular interaction capacities of a group of compounds towards their target structure. The pharmacophore can be considered as the largest common denominator shared by a set of active molecules*.”

We used a pharmacophore-based approach using the HipHop/Catalyst protocol implemented in Discovery Studio. HipHop mainly focuses on the critical common features present in the set of odorants. The terms “pharmacophore model”, “pharmacophore”, “model” and “hypothesis” are interchangeable and refer to the collection of features necessary for the biological activity of the ligands oriented in a 3D space [41].

Our study was carried out on the following training sets:

1. The three “true odor” subsets, t-STR, t-CAR and t-PNA;
2. The two “mixed odor” subsets, STR-CAR and STR-PNA;
3. The subset “EXP” (experimental blend), which includes Et-iB, Et-M and Al-H.

For each training set, all the molecules were considered “active” (Principal = 2) and were required to map all the features of the generated pharmacophore (MaxOmitFeat = 0). We used four chemical features for pharmacophore generation, hydrophobic feature (Hy), hydrophobic aliphatic feature (Hy-al), hydrogen bond acceptor (HBA), and lipid hydrogen bond acceptor (HBA-lip).

The HipHop protocol with these settings produced the top ten hypotheses (Hypo 01 to Hyp 10) for each training set, the details of which are presented in Table 5. The resulting hypotheses were automatically ranked. The most significant hypothesis, “Hypo1”, has a high rank. All features are mapped, and there were no partial hits for any of the hypotheses. Furthermore, the wider the range between the first and tenth hypothesis, the smaller global reliability of the models.

The 10 generated hypotheses consist of at least three features comprising two or three hydrogen bond acceptors (HBA or HBA-lip) and one or two hydrophobic features (Hy or Hy-al).

The ten Hypo t-STRs are the only hypotheses that have a single HBA/HBA-lip feature. All other hypotheses possess at least two hydrogen bond acceptors, and three HBA-lip features are present in the six most reliable hypotheses of t-CAR. The hypotheses of t-CAR differ from those of t-PNA with respect to the nature of the hydrophobic features. Indeed, the t-CAR hypotheses contain Hy features, while the t-PNA hypotheses include only Hy-al features. Two Hy-al and two HBA/HBA-lip features are present in all the hypotheses of t-PNA and STR-PNA. One Hy-al and two HBA/HBA-lip features are present in both the STR-CAR and EXP hypotheses.

Based both on the rank values and on the range between the 1st and 10th hypotheses, it appears that the t-PNA and t-CAR subsets provided the most reliable hypotheses. Conversely, the EXP subset generated the least reliable hypotheses. The large decrease in the rank values of the t-STR hypotheses also indicates the poor reliability of the models.

**Figure 8:**
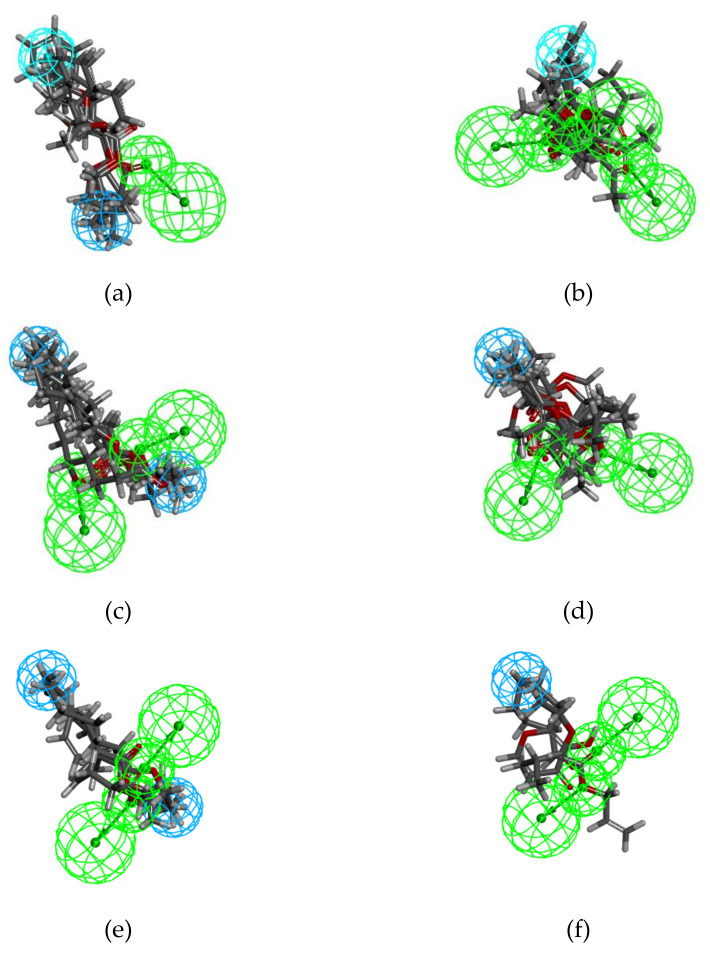
Mapping of the ligands to the chemical features of Hypos_01 generated from the corresponding subsets: (**a**) t-STR; (**b**) t-CAR; (**c**) t-PNA; (**d**) STR-CAR; (**e**) STR-PNA; (**f)** and EXP. The green, cyan and light cyan spheres represent hydrogen bond acceptor (HBA and HBA-lip), hydrophobic aliphatic (Hy-al) and hydrophobic (Hy) features, respectively.

The best hypotheses, models Hypo1, generated for each subset and the mapping of the ligands are displayed in Figure 8:

##### Pharmacophore Comparisons

**Figure 9:**
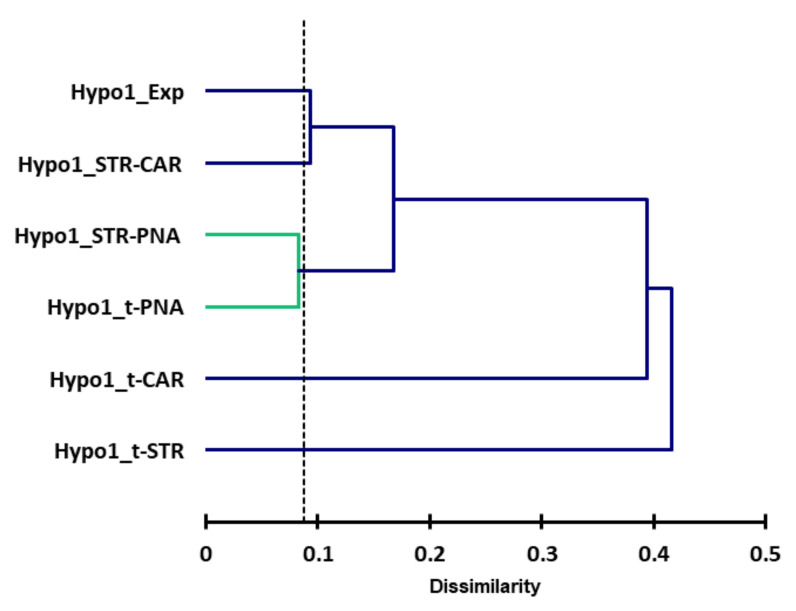
Dendrogram of the odorants obtained by cluster analysis for the ligand-based common feature hypothesis. The proximity matrix is reported in Table A1.

We performed a cluster analysis to evaluate the similarities between the six most reliable hypotheses generated from each subset. The dendrogram (Figure 9) reveals the greatest difference between Hypo1_t-STR and the other hypotheses, while the most similar hypotheses are Hypo1_t-PNA and Hypo1_STR-PNA.

Indeed, the cluster analysis is based on the type and number of features. As observed above, Hypo1_t-STR and Hypo1_t-CAR are the only hypotheses involving one Hy (Z), and Hypo1_t-CAR has no Hy-al (Y) feature. Moreover, unlike all the other hypotheses, which have at least two HBA-lip features, Hypo1_t-STR possesses only one HBA-lip structure. Two Hy-al and two HBA-lip features are present in both Hypo1_t-PNA and Hypo1_STR-PNA.

Nevertheless, comparing the number of hydrophobic and HBA features is not enough to explain the similarities among the hypotheses. Indeed, the distances between the chemical features are needed to elucidate the role of the odorants in olfactory coding. Thus, we observed short distances between a hydrophobic feature and an HBA in several hypotheses; for example, in Hypo1_t-STR, Hypo1_t-PNA and Hypo1_STR-PNA, the distance between the Hy/Hy-al and HBA-lip features is approximately 8 Å (Table 6). Considering how the features in these hypotheses are distributed in 3D space and how the features in two hypotheses overlap in this way is essential.

To compare the geometry of the hypotheses and the distances between the features, it is necessary to map and align the pharmacophores in pairs. For that purpose, we performed several pharmacophore comparisons using the protocol *Pharmacophore Comparison*, which aligns an input pharmacophore to a reference pharmacophore. The root-mean-squared displacement (RMSD) between the matching features and the global RMSD value allows quantitative estimation of the quality of the mapping. Nevertheless, visual observation is crucial for validating the meaning of the mapping.

We focused on several representative mappings based on the cluster analysis of hypotheses and the distances between the features. We first examined the pharmacophores shown to be the closest by cluster analysis (Figure 9).

**Figure 10:**
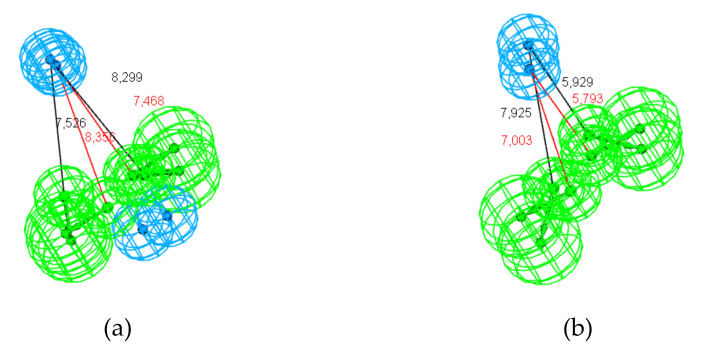

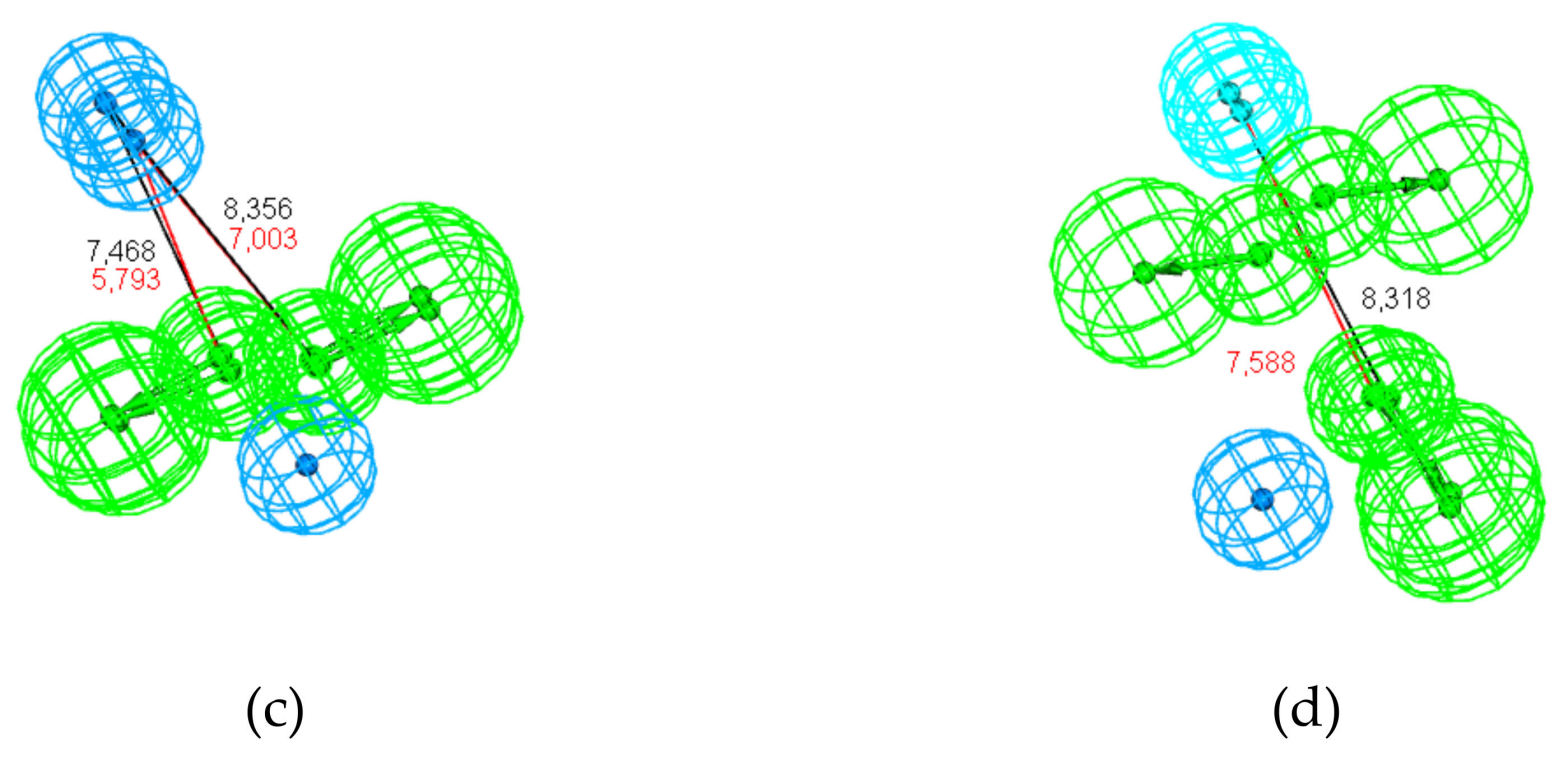
Pharmacophore mapping of the closest hypotheses according to cluster analysis. (**a**) Hypo1_t-PNA and Hypo1_STR-PNA (distances between Hypo1_t-PNA features are shown in black, and distances between Hypo1_STR-PNA features are shown in red); (**b**) Hypo1_STR-CAR and Hypo1_EXP (distances between Hypo1_STR-CAR features are shown in black, and distances between Hypo1_EXP features are shown in red); (**c**) Hypo1_STR-PNA and Hypo1_EXP (distances between Hypo1 STR-PNA features are shown in black, and distances between Hypo1 EXP features are shown in red); and (**d**) Hypo1_t-STR and Hypo1_t-CAR (distances between Hypo1 t-STR features are shown in black, and red distances between Hypo1_t-CAR features are shown in red).

According to the cluster analysis, hypotheses Hypo1_t-PNA and Hypo1_STR-PNA are the only ones that belong to the same cluster. As shown in Figure 10a, despite a poor RMSD value (RMSD = 1.40), the features were satisfactorily mapped, especially the Hy-al2 of each pharmacophore. Although the HBA-lip4 features of t-PNA and STR-PNA only partially overlap, the projections coincide.

In the same way, the cluster analysis also highlighted the small distance between Hypo1_STR-CAR and Hypo1_Exp. The distances between the Hy-al and the HBA-lip features are rather similar for the two models; nevertheless, the mapping is average because the centers and the projection of HBA do not overlap (Figure 10b; RMSD = 1.30).

In addition, a small difference (0.134) was found between Hypo1_STR-PNA and Hypo1_EXP even though they have different numbers of hydrophobic features. The comparison of these two pharmacophores showed substantial overlap between Hy-al2 and Hy-al1 as well as between the HBA-lip features (Figure 10c; RMSD = 0.654). Conversely, there is also a small distance between Hypo1_STR-CAR and Hypo1_STR-PNA (0.157); nevertheless, the RMSD value from the pharmacophore comparison is average (RMSD = 1.189; Figure S2).

In contrast, the greater distances provided by the cluster analysis drawn attention to the pharmacophores Hypo1_t-STR and Hypo1_t-CAR. Nevertheless, the pharmacophore comparison revealed an interesting overlap of their respective Hy and HBA-lip features (RMSD = 0.440). Indeed, the distances from the centers of Hy and the HBA-lip are 8.318 Å and 7.588 Å for Hypo1_t-STR and Hypo1_t-CAR, respectively, which are very close considering the two models (Figure 10d).

The protocol for comparing pharmacophores in pairs is based on the mapping of similar features; the protocol aligns HBA with HBA, HBA-lip with HBA-lip, Hy with Hy, and Hy-al with Hy-la. The mapping of two HBA, or two HBA-lip, is a priority because these features are composed of two parts, the acceptor atom and the projection of the hydrogen bond, and this mapping leads to comparisons with the best RMSD values.

Nevertheless, it is possible to create a tether between two features to connect the location of a feature in the reference pharmacophore to the location of a feature in the input pharmacophore.

**Figure 11:**
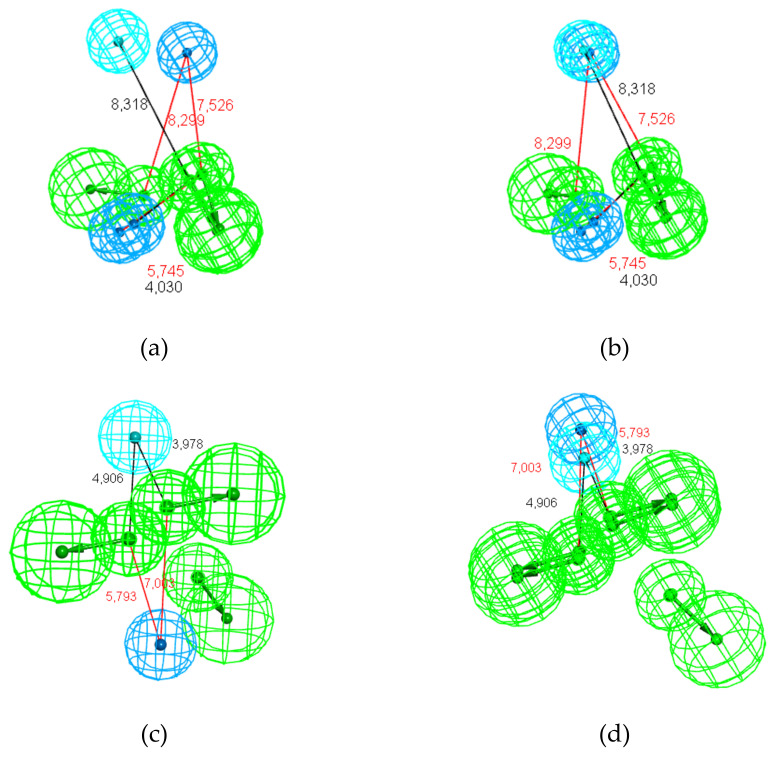
Pharmacophore mappings of the distant hypotheses improved by tethering related hydrophobic features (Hy and Hy-al): (**a**) Hypo1_t-STR and Hypo1_t-PNA (the distances between the Hypo1_t-STR features are shown in black, and the distances between the Hypo1_t-PNA features are shown in red); (**b**) Hypo1_t-STR and Hypo1_t PNA after tethering the hydrophobic features; (**c**) Hypo1_t-CAR and Hypo1_EXP (the distances between the Hypo1 t-CAR features are shown in black, and distances between the Hypo1_EXP features are shown in red); and (**d**) Hypo1_t-CAR and Hypo1_EXP after tethering the hydrophobic features.

The distance values reported in Table 6 suggest possible mapping between Hy and Hy-al features because some distances between Hy/Hy-al and HBA/HBA-lip features are common among several pharmacophores. In that way, we performed a pharmacophore comparison by tethering Hy of Hypo1_t-STR and Hy-al2 of Hypo1_t-PNA. The resulting map, displayed in Figure 11b, shows satisfactory overlap of all the hydrophobic features. However, the short distances between the hydrophobic and HBA spheres are unrealistic for a common receptor site. Conversely, connecting Hy of Hypo1_t-STR to Hy-al1 of Hypo1_t-PNA did not provide a satisfactory result (RMSD = 3.1, Table S5 and Figure S4).

Another example of a better result achieved by using a tether is in the pair of pharmacophores Hypo1_t-CAR and Hypo1_EXP (Figure 11c,d). These two hypotheses differ both in the nature of the hydrophobic features and in the number of HBA-lip features. Nevertheless, the pharmacophore comparison revealed good mapping (RMSD = 0.038). Indeed, as shown in Figure 11c, the two HBA-lip features in each pharmacophore perfectly overlapped. However, there is no overlap between Hy-al (Hypo1_t-CAR) and Hy (Hypo1_EXP). Thus, connecting Hy1 of Hypo1_t-CAR and Hy-al1 of Hypo1_EXP led to satisfactory mapping, as shown in Figure 11d (RMSD = 0.81).

**Figure 12:**
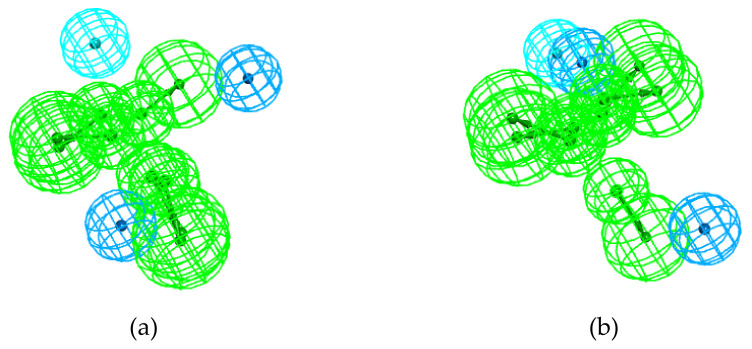
Pharmacophore mappings of Hypo1_t-CAR and Hypo1_t-PNA (**a**) without a tether and (**b**) after tethering hydrophobic features Hy (Hypo1_t-CAR) and Hy-al1 (Hypo1_t-PNA).

A similar procedure was applied to the pharmacophores Hypo1_t-CAR and Hypo1_t-PNA. Without a tether, the mapping involved only the HBA-lip feature (Figure 12a, RMSD = 0.88). Creating a tether between Hy1 of Hypo1_t-CAR and Hy-al1 of Hypo1_t-PNA allowed us to obtain a partial overlay of the hydrophobic features (Figure 12b, RMSD = 1.52), giving an acceptable but average result due to the partial overlap of the hydrophobic features and the divergence in the offset position and direction of the HBA-lip features. Another possible tether involving Hy1 of Hypo1_t-CAR and Hy-al2 of Hypo1_t-PNA led to a worse result regarding both the mapping of the hydrophobic features and those of the HBA-lip features. (Figure S4).

Other cases have been identified:

1. Hypo1_t-STR and Hypo1_STR-CAR: In the absence of a tether, there is partial overlap between the two Hy-al features (Figure S2). Using a tether led to good mappings both between the hydrophobic features and between the HBA-lip features (Figure S3);
2. Hypo1_t-STR and Hypo1_STR-PNA: In the absence of a tether, only one of the two Hy-al features of Hypo1_STR-PNA was mapped, as was one of the HBA-lip features (Figure S2). Using a tether, the two Hy-al features of Hypo1_STR-PNA were mapped with hydrophobic features of Hypo1_t-STR. Nevertheless, there is a deviation between the origins and projections of HBA-lip, and they show only partial overlaps (Figure S3);
3. Hypo1_t-CAR and Hypo1_STR-CAR: In the absence of a tether, two HBA-lip features were mapped, but the Hy-al of Hypo1_STR-CAR overlaps with the projection sphere of one of the three Hy-al features of Hypo1_t-CAR, which is unrealistic with regard to a possible common binding site (Figure S2). Using a tether allows overlap between the hydrophobic spheres and between the two HBA-lip features (Figure S3);
4. Hypo1_t-CAR and Hypo1_STR-PNA: In the absence of a tether, the two HBA-lip features of Hypo1_STR-PNA perfectly match two of the HBA-lip features of Hypo1_t-CAR, but there is no overlap between the hydrophobic features (Figure S2). As in the case of the mapping of Hypo1_t-CAR and Hypo1_t-PNA, the Hy of Hypo1_t-CAR may be connected to Hy-al1 or to Hy-al2 of Hypo1_STR-PNA. Again, only the first option provided an acceptable result, resulting in good overlap of both the hydrophobic features and the HBA-lip features (Figure S3). The alternative, involving a tether between Hy of Hypo1_t-CAR and Hy-al2 of Hypo1_STR-PNA, provides little overlap between these two features (Figure S4).

A visualization of all the pharmacophore comparisons is displayed in Figures S2–S4. The RMSD values of the comparisons are available in Table A2**.**

## 3. Discussion

We undertook this study to identify the significant characteristics, odor quality or molecular properties and features that could explain the configural processing of a well-known binary mixture perceived with a pineapple-like odor. For this purpose, in addition to the two odorants involved in this mixture (Et-iB and Et-M) and a reference odorant (Al-H), we examined the largest StCaPi-set, which includes molecules with either the odor of the mixture components, “strawberry” (STR) and “caramellic” (CAR) or the target odor of the mixture and reference molecule, “pineapple” (PNA). Our study consisted of two parts: (i) a study of the associations of odor notes carried by the molecules in the StCaPi-set using a network and (ii) an analysis of the structural properties of these molecules by a statistical approach conducted on five basic molecular descriptors and a pharmacophore approach.

The results provided by these approaches can be highlighted by several main points.

The main association observed in the StCaPi-set is STR-CAR, and this subset includes nine molecules. Nevertheless, four molecules in the STR-CAR subset are artificial maltol derivatives. Thus, the STR-CAR odor association would include only five nature-identical molecules.

Four molecules showed the STR-PNA association and, of these, three are of a natural origin. Another STR association is STR-“apple”, which was found in four molecules, two of which are natural.

Thus, even considering the natural origin of the molecules, the STR-CAR odor association remains the most frequent, closely followed by STR-PNA. In addition, the network of odors suggested that numerous associations characterize the STR notes in various ways.

Hence, STR seems to be a difficult odor to define because no molecule is simply described with an STR. Strawberry is a very widespread fruit worldwide, but its odor is one the most complex natural odors, and thus it is difficult to clearly describe [42]. The case of Et-iB, which is the STR component in the target mixture, is an interesting example. Indeed, this molecule is not described with an STR note in the Flavor-Base (“*sweet, ethereal, fruity rum-like odor and taste; apple notes*”). However, when Et-iB was subjected to experimental sensory evaluations (by a French panel), it was perceived with a strawberry-like odor [43]. Nonetheless, Et-iB is described as “*rubber, alcoholic, ethereal, strawberry, sweet, fusel, fruity, rummy*” in the FlavorDB [34]. This means that the odor notes “sweet”, “ethereal”, “fruity” and “rum/rummy” are common to these two descriptions, which differ in their “apple” and “strawberry” notes. In addition, “rubber” and “fusel” are specific to the FlavorDB description, but “alcoholic” also refers to “rum”. An “ethereal odor” was also reported by Arctander (“*diffusive sweet ethereal, fruity odor, milder and sweeter, more floral & less fruity than the n-butyrate*” [44]). Interestingly, “winey” and “pineapple” are in the same class as our previously achieved Kohonen classification of odor notes (SOM cl-3m7 × 7) [28].

The examination of the molecular structures highlighted the specificities associated with the different molecules and odors groups of the StCaPi-set odorants.

Et-iB is the smallest molecule in the STR subset. Looking at a series of homologous esters, we observed that methyl isobutyrate has a “pineapple” note (“*fruity, apple-pineapple-apricot-rum like odor*”, while ethyl 2-methylbutyrate is described as “*strong, green, fruity, apple odor and taste; also some strawberry notes*” [33]. Therefore, a small chemical modification alters the odor profile, namely, replacing an ethyl group with a methyl group (decreasing the molecular mass) leads to a “pineapple” note, and adding a methyl group (increasing the molecular mass) increases the STR odor typicality.

The number of rings is indicative of the structural diversity of the STR molecules (Figure 7). Most of the subsets are characterized by a specific number of acyclic or cyclic structures: 85% acyclic structures for PNA molecules and 55% and 89% monocyclic structures for s-CAR (71% monocyclic structures in t-CAR) and STR-CAR, respectively. Conversely, there are almost equal numbers of acyclic, monocyclic and bicyclic STR molecules (four acyclic, three monocyclic and three bicyclic). The bicyclic molecules are naphthyl isomers (naphthyl butyl ether and naphthyl isobutyl ether; C_14_H_16_O) and ethyl methylphenylglycidate (C_12_H_14_O_3_), and these three molecules are in the t-STR subset. Interestingly, the s-CAR subset contains only one bicyclic species, ethyl phenylglycidate (C_11_H_12_O_3_). The two molecules differ only by the methyl group on the alpha carbon of the epoxide (Table S2). We considered ethyl phenylglycidate a CAR molecule, despite its odor description mentioning a “cooked strawberry” note, which differs from a simple “strawberry” odor. The effect of this minor structural variation on odor quality is indicative of the structural similarities between STR and CAR molecules.

Conversely, all the STR-CAR molecules except one have monocyclic structures derived from maltol or furan. Numerous CAR molecules are also derived from maltol and furan, including Et-M. This molecule belongs to both the t-CAR and EXP subsets. The “caramellic” note of Et-M is reported in several odor descriptions (“*sweet, fruity-caramellic cotton candy odor; fruity preserve taste*”, [33]), including that from The Good Scents Company [45] (“*odor sweet caramel jam strawberry cotton candy*”. Conversely, “caramellic” does not appear in the description of Et-M in Arctanders’ book, which describes it as “*intensely sweet, fruity-bread like, pleasant odor of immense tenacity*” [44]; nevertheless, “bread” is frequently associated with “caramellic” (nearly 50% of “bread” occurrences, Table S1).

The strawberry furanone is another case of such an ambiguity. This monocyclic molecule is considered to substantially influence the odor of strawberry fruit [42]. However, the aroma description is complex and has stronger caramellic and cooked odors than it does fresh fruit odors (“*fruity, caramelized pineapple-strawberry odor & taste; roasted*” [33]; “*intense caramellic, fruity, jam like odor with some resemblance to the odor of maltol; also reminiscent of cooked pineapple”* [44]).

The STR-PNA molecules are acyclic esters with saturated, branched and/or unsaturated chains of 7 or 8 carbons, except for ethyl cis-4-decenote (C_12_H_22_O_2_), which is larger than the other compounds. Note that, unlike ethyl cis-4-decenoate (“*fruity, slight floral, pineapple-pear, peach & strawberry notes*”), ethyl trans-4-decenoate does not have an STR note (“*fatty, waxy, green, pineapple and pear-apple notes*”). Additionally, ethyl 5-hexenoate (*“sweet juicy, fruity, pineapple, green; ripe jammy strawberry, apple notes”*) has a “strawberry” nuance but as “ripe jammy strawberry”, and consequently, it was not included in the STR-PNA subset.

These examples and observations concerning all the STR molecules highlight the ambiguous chemical space of their structures and the diversity in their odors. However, the molecules in STR-CAR and STR-PNA meet the criterion of the structural properties of CAR and PNA, respectively.

In contrast to the structural diversity of the STR molecules, the CAR and PNA subsets are more homogeneous in their structural properties. This is true for both the “simple” and “true” subsets as well as the “mixed” subsets, as shown above.

The CAR and PNA molecules have significantly different properties. Moreover, these two odor notes are very rarely both present in the same odor description. We identified a unique case, alpha-furfuryl pentanoate, where both these notes appear in the odor description. Notably, the CAR-PNA association exists in several descriptions concerning flavor but not orthonasal perceptions. Alpha-Furfuryl pentanoate has both a furan and an ester chain. Furan moieties are commonly found in CAR molecules, while numerous aliphatic esters are associated with the “pineapple” note. Several other furfuryl ester derivatives, such as ethyl furylpropanoate (“*fruity, green, woody, unripe fruit; pineapple, chamomile-like*”), 2-furylmethyl decanoate (“*waxy-fatty, somewhat caramel*”), and 2-furylmethyl hexanoate (“*green, fatty, musty, waxy odor; green fruity taste; somewhat caramellic*”), have PNA or CAR odors. However, the CAR note appears to be faint, while the PNA note is quite noticeable, which suggests that the chain is more important than the furfuryl ring in the odor qualities of these compounds.

The results of the pharmacophore study highlighted complementary findings. Regarding the qualitative side of the most reliable hypotheses, several similarities have been identified based on the nature of the related features. The pair-by-pair comparisons between pharmacophores reinforced the similarities and differences among the groups of molecules.

A majority of models contain at least one Hy-al feature and two close HBA-lip features linked to ester functions, and the exceptions to this were Hypo1_t-STR (1 Hy, 1 Hy-al, and 1 HBA-lip) and Hypo1_t-CAR (1 Hy and 3 HBA-lip). This specific composition makes these two models unique relative to all others. The t-CAR models were generated from molecules characterized by high oxygen contents and the absence of aliphatic carbon chains. The t-STR models were generated from diverse structures with few common features. Unsurprisingly, the t-STR model incorporates the characteristics of both the t-CAR model (Hy feature) and the PNA model (Hy-al feature).

The distances between the hydrophobic features and the HBA-lip centers are obviously crucial for achieving satisfactory overlap among the models. It is important to consider that both Hy and Hy-al define the hydrophobic zones regardless of the structural specificity of the related chemical groups. In other words, both a flexible chain and a ring can adopt a similar 3D shape and interact in the same way with a receptor site [46,47]. Considering this viewpoint, we obtained the satisfactory overlays of Hypo1_t-STR and Hypo1_t-PNA and of Hypo1_t-CAR and Hypo1_EXP (Figure 11b,d). Nevertheless, the mappings of t-CAR and t-PNA generated with a tether between Hy of the t-CAR model and Hy-al of the t-PNA model led to only an average level of overlap (Figure 12, Figures S3 and S4). This result suggests that there is a notable difference between the CAR and PNA molecules.

**Figure 13:**
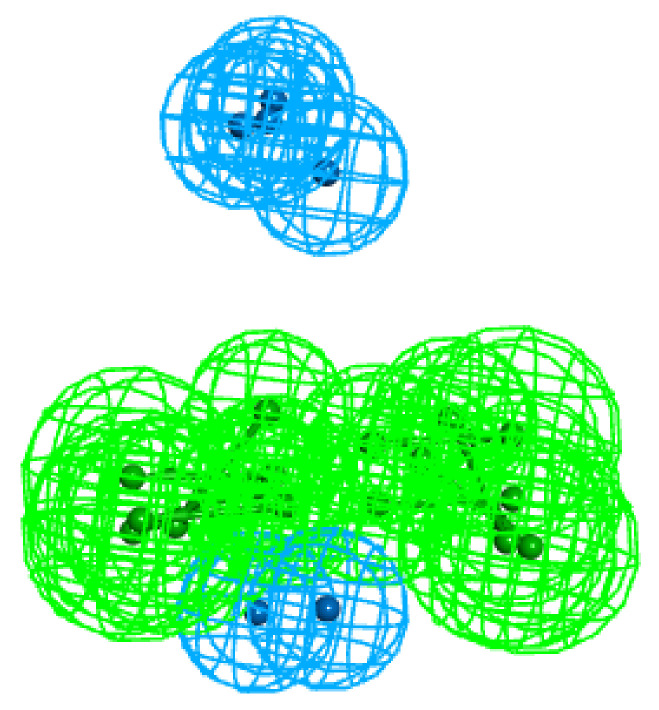
Pharmacophore overlap of the Hypo1_t-PNA, Hypo1_STR-CAR, Hypo1_STR-PNA and Hypo1_EXP models.

The examination of the “mixed odor” models generated from the subsets STR-CAR and STR-PNA provides additional information. The STR-PNA model is nearly identical to the t-PNA model (Figure 9). The two models involve the same features, and they are almost equally spaced (Table 6 and Figure 10a). Thus, the molecules in the STR-PNA subset can be regarded as having the same spatial characteristics as the molecules in the t-PNA subset. In addition, the STR-CAR model presents characteristics similar to those of the EXP model (Figure 10b). Furthermore, the spacing between Hy-al and HBA-lip in the EXP model is on the same order as those in the t-PNA and STR-PNA models. As displayed in Figure 10c in the case of the STR-PNA and EXP models, there is good mapping between the two HBA-lip and one Hy-al feature of the STR-PNA model. As a consequence, the four models display rather good overlap, as shown in Figure 13:

This overlap may be considered a consequence of structural similarities among the molecules in t-PNA and t-STR, as well as the molecules in STR-CAR. In this way, the STR-CAR molecules seem to “resemble” both the t-PNA/STR-PNA and the t-CAR molecules. Notably, the EXP model has a rather poor composition (1 Hy-al and 2 HBA-lip), as well as poor significance related to the range values (Table 5). This is partially because it was generated from a smaller number of molecules but, more importantly, due to the diversity of their structures. Nevertheless, the model was generated and provided 10 hypotheses, which is very rarely the case for unstable models. This fact alone indicates that the three molecules in the EXP subset share a common key structure. Moreover, the mapping of the Hypo1_t-PNA, Hypo1_STR-CAR, Hypo1_STR-PNA and Hypo1_EXP models suggests that this key structure is also shared by the molecules in the t-PNA, STR-CAR and STR-PNA subsets.

## 4. Materials and Methods

### 4.1. Data Preparation

We selected the molecules for the training sets based on their odor notes. The molecules were extracted from the large database that we previously used for multivariate statistical analysis [28] and was built from the 9th version of Flavor-Base [33]. This database, called FB-3508, encompasses 3508 odorants and 251 odors as a binary matrix (1 when the odor note appears in the odor description, 0 otherwise). The selected training set (StCaPi-set) encompasses 293 odorant molecules and 112 odors as a binary matrix. The StCaPi-set finally encompasses 298 structures considering the isomers of five odorants.

### 4.2. Network of Odor Visualization

The study of the network of odor notes first required the calculation of the cooccurrences using the 112 × 112 square matrix of odor notes, and this was conducted using R version 3.0.1 [28,48]. In the cooccurrences matrix, the off-diagonal terms are the number of cooccurrences of the two odor notes in an odorant description, while the diagonal terms are the number of all occurrences of each odor note.

The square matrix was transformed into a two-way data table using Statistica TIBCO Software Inc. [49]. Cytoscape [50] was used to build a network of the links among odor notes.

### 4.3. Statistical Analysis Based on Molecular Properties

The molecular properties were calculated using Discovery Studio 4.5, BIOVIA [51] running on Windows 7 for PC. The odor molecules were gathered in an .sd file that was used to calculate the following molecular properties:

1. 1D properties, Molecular Formats:
  - Canonical_Smiles: A form of SMILES (textual representation of molecular data) that is independent of how the molecule is drawn;
  - ChemicalName: The systematic name for the chemical compound generated according the IUPAC rules;
  - InChI: The IUPAC unique identifier (capable of uniquely representing a chemical substance). It is derived from a structural representation of that substance that is independent of the way the structure is drawn.
2. 2D properties:
  - AlogP98: Log of the octanol-water partition coefficient using Ghose and Crippen’s method [52];
  - Apol: Polarizability descriptor, i.e., the sum of the atomic polarizabilities;
  - Molecular_Formula: The molecular formula is formatted according to the following rules: carbon first, hydrogen second, all remaining elements in alphabetical order;
  - Molecular_Weight: The sum of the atomic masses. The isotope average is used for each atomic mass.
3. Molecular Property Counts:
  - Num_Rings: Base rings, defined as the number of rings in the smallest set of smallest rings.
4. Topological Descriptor:
  - PHI: Molecular Flexibility (Kappa Shape Index). This descriptor is based on structural properties that prevent a molecule from being “infinitely flexible”, which is represented by an endless chain of C(sp3) atoms. The structural features considered to prevent a molecule from attaining infinite flexibility are (i) fewer atoms, (ii) the presence of rings, (iii) branching, and (iv) the presence of atoms with covalent radii smaller than those of C(sp3).
5. 3D properties:
  - Molecular_3D_PolarSASA: The polar solvent accessible surface area for each molecule was calculated using a 3D method. Atoms that are considered polar are N, O, P, S, the hydrogens attached to them, and any atom with a formal charge.

The 2D and 3D molecular properties were used for the statistical analysis.

Microsoft^®^ Excel 2010/XLSTAT©-Pro [53] (2019.1.2, Addinsoft, Inc., Brooklyn, NY, USA) was employed for the statistical evaluations.

Three statistical tests, namely, descriptive statistics, normality tests, and a nonparametric test (the Kruskal–Wallis test), were performed to examine the molecular descriptor values. For the nonparametric Kruskal–Wallis test, multiple pairwise comparisons were performed using Dunn’s procedure. The significance level α was set to 0.05 (*p* < 0.05).

### 4.4. Computational Chemistry

The computational analyses were conducted using Discovery Studio 4.5, BIOVIA [51] running on Windows 7 for PC.

#### Common Feature Pharmacophore Generation

Pharmacophores were generated using the HipHop/Catalyst protocol implemented as the “Common Feature Pharmacophore Generation” protocol in Discovery Studio 4.5 [41,51]. A maximum of 250 conformers were generated in a range of 0–20 kcal/mol (BEST conformer generation protocol [54]). The maximum number of generated hypotheses for each run was set to 10.

In our study, the pharmacophoric features considered are hydrogen bond acceptors (HBA features), lipid hydrogen bond acceptors (HBA-lip features), hydrophobic regions (Hy features) and hydrophobic aliphatic regions (Hy-al features).

- HBA: matches electronegative atoms that have a lone pair and a charge less than or equal to zero (sp^3^ oxygens or sulfurs and sp or sp^2^ nitrogens); does not match basic amines;
- HBA-lip: the same as HBA except that it includes basic nitrogens;
- Hy: matches groups of contiguous sets of atoms (such as methyl, isopropyl, cycloalkyl, and phenyl);
- Hy-al: the subset of Hy that includes only aliphatic atoms.

Due to the relatively small size of the odorants (74 < MW < 260), the parameter “Minimum Interfeature Distance” was decreased from its default value of 2.97 Å to 0.5 Å.

In the advanced parameters, the minimum number of feature points (Minimum Feature Points and Minimum Features in Moderately Active) were both set to 2. Default values were used for the other parameters.

Because the activities are unknown and probably vary according to the different target ORs of each odorant, all the molecules were regarded as reference “Active” molecules, and the parameter “Principal”, which indicates the activity level of the molecule, was set to 2. The maximum omitted features parameter (“MaxOmitFeat”) specifies how many features the generated pharmacophore is allowed to miss for each molecule:

- If MaxOmitFeat = 0, all features must map to this molecule;
- If MaxOmitFeat = 1, all except one of the features must map to this molecule;
- If MaxOmitFeat = 2, no features need to be mapped to this molecule.

For each run, MaxOmitFeat was first set to 0 for all molecules. The fit value of each molecule reflects the quality of its mapping, and a greater value of best fit indicates that the molecule is a better fit for the hypothesis.

The pharmacophores were compared using the “Cluster Pharmacophores” protocol and the “Pharmacophore Comparison” protocol.

Cluster analysis allows us to evaluate the similarities between the pharmacophore models in terms of the nature and location of the chemical features. The Cluster Pharmacophores protocol calculates the distance between each pair of pharmacophores. This distance is a function of the number of common pharmacophore features and the root-mean-squared displacement (RMSD) between the matching features. The proximity matrix is clustered and is presented as a dendrogram.

The Pharmacophore Comparison protocol allows the mapping and alignment of two pharmacophores; an RMSD value is reported for the matching pharmacophore features. The “Best Mapping Only” parameter was used for the comparisons. The results obtained from the various analyses performed in this work support the following statements:

- CAR and PNA molecules have almost nothing in common. Very few molecules carry both CAR and PNA notes. Each of the two groups of molecules has rather homogeneous molecular properties. The structural investigations through the statistical study of the molecular properties as well using the pharmacophore approach agree that there is a general lack of common characteristics;
- In addition, STR molecules do not share clear common characteristics, neither in their odor descriptions nor in their structural features. These molecules “look like” CAR or PNA molecules depending on the examined property (for example, hydrophobicity vs. flexibility). Most STR-CAR molecules are cyclic, similar to several CAR molecules, while STR-PNA molecules are esters, as are numerous PNA molecules.

Several examples highlight the unclear distinction between the CAR and STR molecules. The results suggest that the STR odor does not intrinsically exist but has an ambiguous character involving an amalgam of other odors. That could be the key advantage that allows it to serve as a bridge between incompatible odorants, such as, in this case, CAR and PNA. STR seems to have a central role because of its “multivalent” character, which would allow it, by association with another odorant, to reveal another odor note not just for this peculiar aroma-blending mixture, but perhaps for more common blends.

The pharmacophore approach allowed the identification of several peculiarities in the spatial distribution of the molecular features. The main characteristics are described in Figure 13. There are two pairs of adjacent HBA-lip and hydrophobic features; one hydrophobic feature is less than 6 Å away from the HBA-lip centers, and the other is 6–8 Å away. The two hydrophobic features are approximately 10 Å apart. Not all the models have two Hy or Hy-al features; nevertheless, the hydrophobic features of the t-STR, t-CAR and EXP models meet one of the distance criteria.

## 5. Conclusions

The stated attributes allow the drawing a “Portrait Robot” of the STR + CAR ≡ PNA” aroma-blending mixture. By attributing A to STR, B to CAR and C to PNA, the aroma blending can be generalized and summarized as follows:

- The chemical structures of B and C are noticeably different, and B and C have either no, or only a few, common features. The major odor notes of B and C can be clearly determined, and their primary notes are quite frequent in the odorant descriptions of a large database. The “B” and “C” odors are not directly connected in a network of numerous odor notes but have numerous common links;
- The molecules sharing the “A” odor have diverse chemical structures, with some comparable to those of B or C molecules. The “A” odor is uncommon among odorants. This odor is frequently present in odor descriptions, but never alone in any description, with the odors of B and C being its most frequent associations;
- Despite the differences and structural variations in the molecules carrying the odors of A, B or C, the spatial distribution of their chemical features meets the same distance criteria. This point suggests that molecules A, B and C could share one or more common OR target(s), and they could interact with these target(s) through diverse roles, such as agonist, antagonist, and inverse agonist.

Such a “Portrait Robot” could obviously be specific to the “STR + CAR ≡ PNA” blend. Nevertheless, one assumption could be that these characteristics would be shared by other aroma blending. If this supposition turns out to be true, the identification of sets of three molecules with similar characteristics would provide odorant candidates, and ultimately help in the design of new aroma-blending mixtures.

## Figures and Tables

**Table 1:** Descriptions of odorants in the “true odors” subsets.

Odorant Name	Odor Description [33]
**t-STR Subset**	
Ethyl methylbutyrate	Strong, green, fruity, apple and taste; some strawberry notes
Ethyl 4-methylpent-3-enoate	Fruity, green, apple, berry, strawberry, mixed fruit
Ethyl methylphenylglycidate	Sweet, fruity-strawberry, candy-like
Fraistone	Fresh, sweet-fruity notes reminiscent of apple and strawberry
Naphthyl butyl ether	Sweet tenacious fruity and floral note reminiscent raspberry and strawberry
Naphthyl isobutyl ether	Sweet, strawberry-fruity, neroli-like
Phenylpropyl isovalerate	Fruity (strawberry-prune)
**t-CAR Subset**	
Benzyl levulinate	Sweet caramellic-fruity
Cyclotene acetate	Caramellic, somewhat fruity
Dihydrodihydroxymethylpyranone	Weak caramellic, sugar notes
Et-M	Sweet, fruity-caramellic cotton candy
Ethyl pyruvate	Sweet, fruity-caramellic
Propyl levulinate	Sweet, slight fruity, caramellic
Sotolon	Powerful caramel aroma
**t-PNA Subset**	
Allyl cyclohexanebutyrate	Sweet-fruity, pineapple
Ethyl cyclohexanepropionate	Strong, sweet, fruity, pineapple
Ethyl 3-methylpentanoate	Fruity, pineapple
5-Hexenyl butyrate	Green, fruity, pineapple
Isopropyl hexanoate	Sweet, fruity pineapple-like
Methyl cis-3-hexenoate	Fruity-green, pineapple

**Table 2:** Molecular descriptor values of the three molecules used in the experimental blending, the unique molecule CAR-PNA, and strawberry furanone (“caramelized pineapple-strawberry”).

Current Name	MW	ALogP98	Apol	PHI	3D_PolarSASA
Et-M	140.137	0.301	5365.84	1.98809	99.273
Al-H	156.222	2.673	5873.68	7.04851	48.001
Et-iB	116.158	1.499	4019.26	3.44283	39.429
alpha-Furfuryl pentanoate	182.216	2.365	6905.62	4.25708	69.43
Strawberry furanone	128.126	0.113	4537.94	1.38454	110.066

**Table 3:** Statistical parameters of the Kruskal–Wallis test on the distribution of the molecular descriptors for the odor subsets.

Subsets Comparisons	Statistical Parameters ^1^	Molecular Descriptors				
		MW	ALogP98	Apol	PHI	3D_PolarSASA
All subsets	K (Observed value)	44.583	126.308	37.971	147.884	124.036
	K (Critical value)	14.067	14.067	14.067	14.067	14.067
	*p*-value (one-tailed)	< 0.0001	< 0.0001	< 0.0001	< 0.0001	< 0.0001

^1.^ Degree of freedom = 7 for the comparisons of 8 subsets, 2 for the three comparisons.

**Table 4:** Multiple pairwise comparisons between the odor subsets using Dunn’s procedure.

Odors Subsets	Descriptor	Subsets	Sum of Ranks	Mean of Ranks	Groups		
	MW	s-CAR	18140.000	124.247	A		
		t-CAR	911.500	130.214	A	B	
		STR-PNA	585.500	146.375	A	B	
		STR-CAR	1597.500	177.500	A	B	
		s-PNA	23661.000	187.786		B	
		t-PNA	1392.500	198.929		B	
		s-STR	2210.000	221.000		B	
		t-STR	1588.000	226.857		B	
	ALogP98	t-CAR	333.000	47.571	A		
		STR-CAR	583.000	64.778	A		
		s-CAR	15840.500	108.497	A	B	
		s-STR	1972.500	197.250		B	C
		t-STR	1417.500	202.500		B	C
		STR-PNA	827.500	206.875		B	C
		s-PNA	27490.500	218.179			C
		t-PNA	1621.500	231.643			C
	Apol	s-CAR	18645.000	127.705	A		
		t-CAR	902.000	128.857	A	B	
		STR-PNA	554.500	138.625	A	B	
		STR-CAR	1614.500	179.389	A	B	
		t-PNA	1276.000	182.286	A	B	
		s-PNA	23253.000	184.548		B	
		t-STR	1581.000	225.857		B	
		s-STR	2260.000	226.000		B	
	PHI	STR-CAR	747.500	83.056	A		
		t-CAR	634.000	90.571	A		
		s-CAR	14926.500	102.236	A		
		t-STR	918.000	131.143	A	B	
		s-STR	1480.000	148.000	A	B	
		STR-PNA	897.000	224.250	A	B	
		s-PNA	28831.000	228.817		B	
		t-PNA	1652.000	236.000		B	
	3D_PolarSASA	t-STR	543.500	77.643	A		
		STR-PNA	343.500	85.875	A		
		t-PNA	686.500	98.071	A		
		s-PNA	12721.500	100.964	A		
		s-STR	1214.000	121.400	A	B	
		s-CAR	30601.000	209.596		B	
		STR-CAR *	2167.000	240.778			
		t-CAR *	1809.000	258.429			

* Groupings were not performed because the significance of the differences is not transitive in this particular case.

**Table 5:** Details of the features and ranks of the top ten hypotheses generated using HipHop for each training set.

“True Odor” Subsets						
Subset	t-STR		t-CAR		t-PNA	
Direct Hit ^a^	1111111		1111111		1111111	
Hypo	Features ^b^	Rank ^c^	Features ^b^	Rank ^c^	Features ^b^	Rank ^c^
1	YZH	46.7	ZHHH	57.2	YYHH	72.9
2	YZH	45.7	ZHHH	56.8	YYHH	71.8
3	YZA	45.3	ZHHH	56.6	YYHH	71.7
4	YZA	44.3	ZHHH	56.5	YYHH	71.7
5	YZH	42.5	ZHHH	56.4	YYHA	71.5
6	YZH	42.3	ZHHH	56.0	YYHA	71.5
7	YZH	41.8	ZHHA	55.8	YYHA	71.5
8	YZA	41.1	ZHHA	55.8	YYHH	71.2
9	ZZH	41.1	ZHHA	55.8	YYHH	70.8
10	YZA	40.9	ZHHA	55.4	YYHH	70.4
**“Mixed Odor” Subsets**						
**Subset**	**STR-CAR**		**STR-PNA**		**EXP**	
Direct Hit ^a^	111111111		1111		111	
Hypo	Features ^b^	Rank ^c^	Features ^b^	Rank ^c^	Features ^b^	Rank ^c^
1	YHH	51.1	YYHH	36.5	YHH	17.0
2	YHH	50.2	YYHA	35.7	YHA	16.4
3	YHH	49.9	YYAA	34.9	YHA	16.4
4	YHA	49.3	YYHA	34.8	YAA	15.8
5	YHA	49.3	YYHH	34.7	YHH	15.4
6	YHH	49.0	YYHA	34.6	YHA	14.8
7	YHA	48.4	YYHH	34.2	YHA	14.8
8	YHA	48.4	YYHH	33.9	ZHH	14.6
9	YHA	48.1	YYHA	33.9	YHA	14.5
10	YHA	48.1	YYHA	33.9	YAA	14.2

^a.^ Same direct hits for all hypotheses; no partial hits for any of the hypotheses. ^b.^ A: HBA, H: HBA-lip, Z: Hy, Y: Hy-al. ^c.^ The higher the ranking score, the lower the probability of chance correlation is. The best hypotheses have the highest values.

**Table 6:** Distances between features of the best significant hypotheses Hypo1_t-STR, Hypo1_t-CAR, Hypo1_t-PNA, Hypo1_STR-CAR, Hypo1_STR-PNA and Hypo1_EXP.

Hypo1	atom1	atom2	Distance (Å)
t-STR	Hy-al1	HBA-lip3	4.03
	Hy2	HBA-lip3	8.318
	Hy-al1	Hy2	9.873
t-CAR	Hy1	HBA-lip2	3.978
	Hy1	HBA-lip3	4.906
	Hy1	HBA-lip4	7.588
	HBA-lip2	HBA-lip3	2.199
	HBA-lip3	HBA-lip4	5.409
	HBA-lip2	HBA-lip4	5.525
t-PNA	Hy-al1	HBA-lip3	2.717
	Hy-al1	HBA-lip4	5.745
	Hy-al2	HBA-lip3	8.299
	Hy-al2	HBA-lip4	7.526
	Hy-al1	Hy-al2	10.669
STR-CAR	Hy1	HBA-lip2	5.929
	Hy1	HBA-lip3	7.925
	HBA-lip2	HBA-lip3	3.473
STR-PNA	Hy-al1	HBA-lip3	3.112
	Hy-al1	HBA-lip4	2.458
	Hy-al2	HBA-lip3	7.468
	Hy-al2	HBA-lip4	8.356
	Hy-al1	Hy-al2	10.129
EXP	Hy-al	HBA-lip2	5.793
	Hy-al	HBA-lip3	7.003
	HBA-lip1	HBA-lip2	2.254

## Supplementary Materials

The following are available online. Table S1. List of the 293 odorants in the StCaPi-set and their odor notes. Table S2. The molecular structures of the 298 compounds in the StCaPi-set and their related molecular descriptor values. Table S3. Descriptive statistics parameters. Table S4. Normality tests for the molecular descriptor distributions. Table S5. Detailed statistical results of the pairwise comparisons. Figure S1. Distributions of the molecular property values for the eight subsets (s-STR, s-CAR, s-PNA, t-STR, t-CAR, t-PNA, STR-CAR, and STR-PNA). Figure S2. Mapping obtained by the Pharmacophore Comparison protocol: visualization of Hypo1 pharmacophores mapped by pairs. Figure S3. Best alternative mapping obtained by the Pharmacophore Comparison protocol using a tether between one Hy and one Hy-al. Figure S4. Lowest alternative mapping obtained by the Pharmacophore Comparison protocol using a tether between one Hy and one Hy-al.

## Appendix A

**Table A1:** Proximity matrix between the most reliable hypotheses obtained by the cluster analysis protocol of the pharmacophores.

Pharmacophore	Hypo1_ t-STR	Hypo1_ t-CAR	Hypo1_ t-PNA	Hypo1_ STR-CAR	Hypo1_ STR-PNA	Hypo1_ EXP
Hypo1_t-STR		0.41565	0.39396	0.38173	0.37536	0.35924
Hypo1_t-CAR	0.41565		0.39382	0.34439	0.33949	0.28393
Hypo1_t-PNA	0.39396	0.39382		0.16128	0.0823	0.16775
Hypo1_STR-CAR	0.38173	0.34439	0.16128		0.15703	0.09312
Hypo1_STR-PNA	0.37536	0.33949	0.0823	0.15703		0.13441
Hypo1_EXP	0.35924	0.28393	0.16775	0.09312	0.13441	

**Table A2:** RMSD values obtained by pharmacophore comparisons by pairs.

Pharmacophores Hypo1_	t-STR	t-CAR	t-PNA	STR-CAR	STR-PNA	EXP
t-STR		0.439746	0.716228 0.937989 * 3.106499 **	1.619884 0.451375*	0.441043 1.254882*	0.977177

t-CAR			0.876108 1.518761 * 2.017727 **	0.856909 1.346555 *	0.058114 0.793919 * 1.388842 **	0.038287 0.811610 *

t-PNA				1.330698	1.400906	1.585165

STR-CAR					1.189394	1.30305

STR-PNA						0.653636

EXP						

* Improved mapping obtained using a tether between the hydrophobic features Hy and Hy-al ** Not suitable mapping was obtained by an alternative tether between the hydrophobic features Hy and Hy-al.
