# Supplementary material for: Exploring the Characteristics of an Aroma-Blending Mixture by Investigating the Network of Shared Odors and the Molecular Features of Their Related Odorants
Source: Molecules. 2020 Jul 2;25(13):3032. doi: 10.3390/molecules25133032 (PMC7411594; doi:10.3390/molecules25133032)
Supplement: Supplementary file 1 [file molecules-25-03032-s001.zip › FigureS2.pdf]

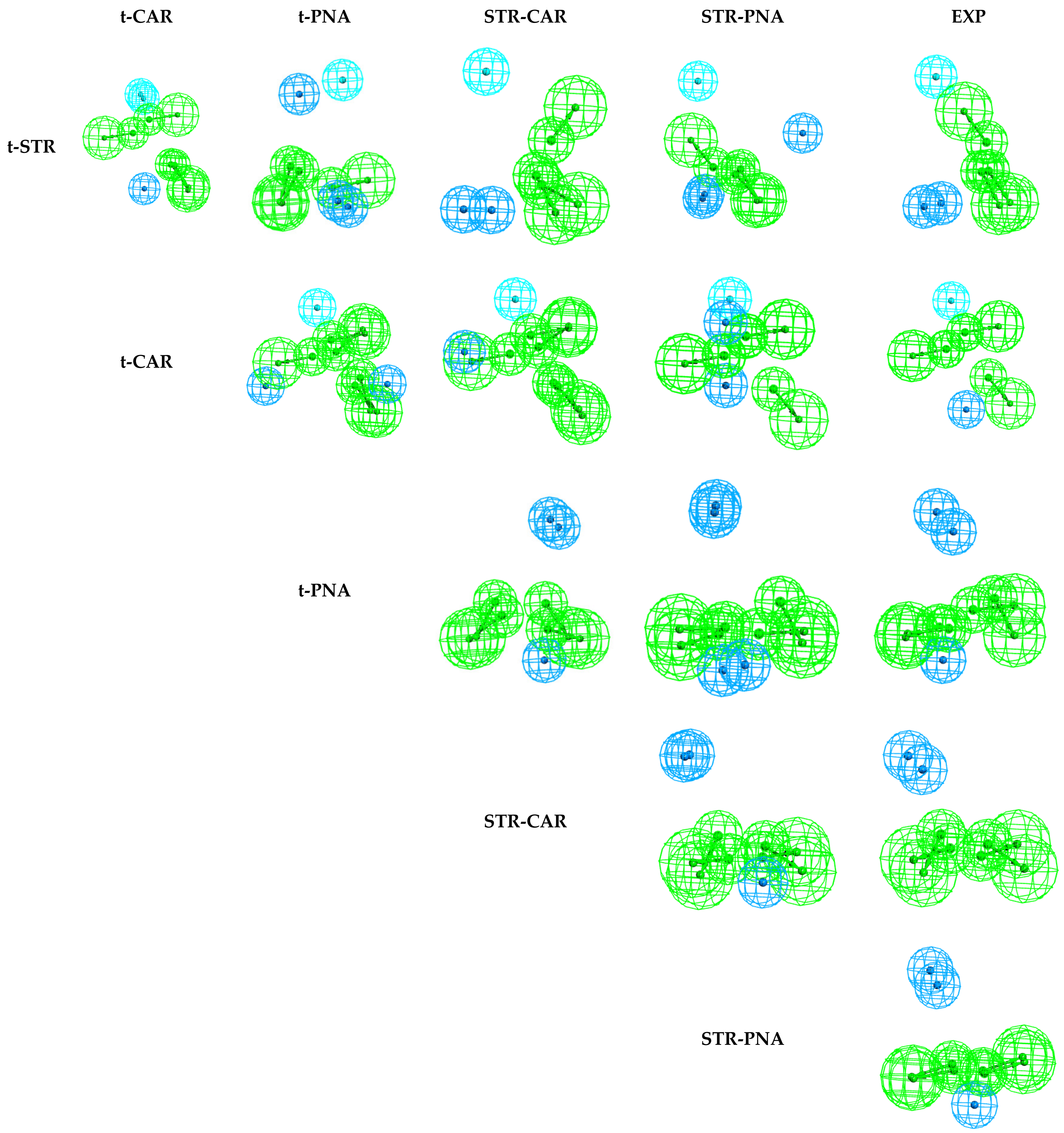

Figure S2. Mapping obtained by the Pharmacophore Comparison protocol: visualization of Hypo1 pharmacophores mapped by pairs.
